# Digital Eversion of a Hollow Structure: An Application in Virtual Colonography

**DOI:** 10.1155/2008/763028

**Published:** 2008-07-22

**Authors:** Jun Zhao, Liji Cao, Tiange Zhuang, Ge Wang

**Affiliations:** ^1^Department of Biomedical Engineering, Shanghai Jiao Tong University, Shanghai 200240, China; ^2^Biomedical Imaging Division, VT-WFU School of Biomedical Engineering and Sciences, Blacksburg, VA 24061, USA

## Abstract

A new methodology is presented for digital eversion of a hollow structure. The digital eversion is advantageous for better visualization of a larger portion of the inner surface with preservation of geometric relationship and without time-consuming navigation. Together with other techniques, digital eversion may help improve screening, diagnosis, surgical planning, and medical education. Two eversion algorithms are proposed and evaluated in numerical simulation to demonstrate the feasibility of the approach.

## 1. INTRODUCTION

Computed tomography (CT), magnetic resonance (MR), or other biomedical imaging modalities are frequently used for inspecting of the inner surfaces of hollow structures such as colon and stomach. For those purposes, many visualization techniques were developed, some of which have been adopted in clinical applications [1–7]. However, with traditional visualization techniques only a very small portion of inner surfaces can be presented in one view, because neither an operator's perspective nor a field of view with virtual endoscopy is unlimited. Hence, the navigation is typically necessary in a visualization session, which is time-consuming, tedious, and error prone particularly when features of interest are hidden behind folds [8, 9].

To overcome the aforementioned difficulties in visualizing anatomical cavities, Wang et al. proposed a method for unraveling the colon based on the electrical field model [10–12]. Subsequently, Zhu et al. presented two algorithms for flattening branched vessels [13]. Other unfolding or flattening methods were also reported [14–19]. With these unwrapping techniques, the entire inner surface of a hollow structure can be mapped into one view. However, such unraveling operations distort original shapes and relative configurations, compromising the intended benefits significantly. Fletcher et al. developed a planar virtual pathology (PVP) method, in which a segment of the colon is cut open to display the luminal surface [20]. The PVP method is geometrically faithful, keeping the relationship between the colon and neighboring organs. Furthermore, this method may display a larger portion of the luminal surface more efficiently than the conventional virtual endoscopy. However, the PVP method only handles one opened segment each time, and needs a series of segments with various cutting planes to examine the whole colon.

In this paper, we present a totally new idea for visualizing the inner surface of a hollow structure digital eversion meaning to turn inside to outside via computer [21, 22]. In other words, the inner surface is converted to the outer surface with the inward normals reserved outward. The primary advantages of digital eversion over conventional virtual endoscopy include the direct visualization of a larger portion of the inner surface and the close correlation to the important features of the global anatomy, without the need for tedious and time-consuming navigation. Digital eversion of a hollow structure is complementary or superior to the digital flattening technique in terms of preserving the involved geometric center, center line, shape, and spatial relationship. Together with other visualization techniques, digital eversion may help improve diagnosis, surgical planning, and medical education.

In Section 2, we present two algorithms for everting the colon as a major application. One algorithm performs eversion along electrical force lines formed by simulated charges on the central colon path. The other algorithm performs eversion along electrical force lines formed by simulated charges on the reference surface of the colon. In Section 3, we describe experimental results to show the feasibility of digital eversion of a colon. In Section 4, we discuss relevant issues and conclude.

## 2. MATERIALS AND METHODS

**Figure 1:**
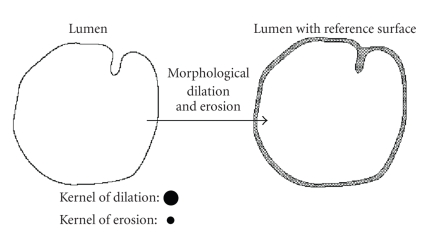
A 2D-illustration of the reference surface generating by mathematical morphology.

After a real eversion of the colon, the mucosal surface becomes the outer surface while the serosal surface becomes the inner surface. Similarly, with our digital eversion, the mucosal surface and serosal surface of the colon are exchanged with respect to the reference surface that is the average of the mucosal and serosal surfaces. The reference surface is quite like a curved “mirror” in the eversion process. Practically, the outer surface of the colon is extremely difficult to be identified due to the low contrast between the colonic wall and pericolonic tissues. Clinically, the detection of polyps and masses on the mucosal surface is of primary interest. Hence, we do not need to be concerned about the serosal surface. Without a knowledge on the serosal surface, the reference surface can still be generated by applying 3D mathematical morphology operations to the colon lumen. Specifically, the colon lumen can be first dilated by a spherical kernel with a relatively large radius, and then eroded with a small spherical kernel if it is necessary. The radius of the dilating kernel should be larger than the sizes of folds and polyps. After these morphologic transforms, the surface wrapping the colon lumen will be relatively smooth, ready to be used for eversion (see Figure 1).

### 2.1. Centerline-based eversion

**Figure 2:**
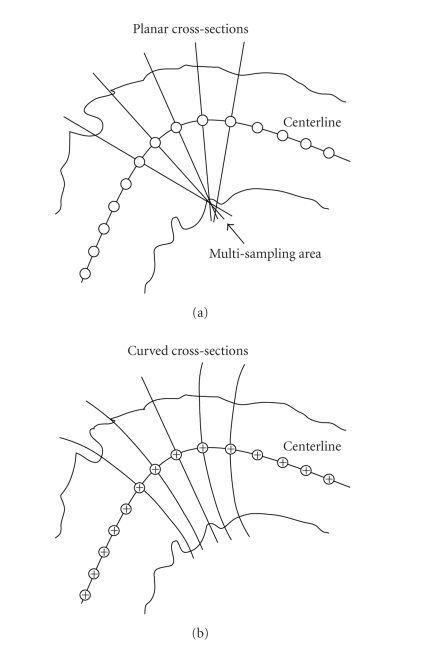
Cross-sections generated for a tubular structure with respect to its centerline. (a) Possible intersections of planar cross-sections perpendicular to the centerline, which may lead to redundant or little sampling of the surface, and (b) curved cross-sections based on an electrical field, in which positive charges are distributed along the centerline.

This centerline-based eversion algorithm is suitable for analyzing tubular structures. The center line of the colon lumen can be automatically extracted, for example, using the distance transform and Dijkstra's shortest path technique [6]. The most straightforward technique for eversion is based on a sequence of planar cross-sections that are orthogonal to the centerline. However, these cross-sections may intersect, which can cause redundant or little sampling of the colon surface (Figure 2(a)) [10, 19].

Wang et al. used a centerline-based method for unraveling the colon [10]. We adapted their idea for the digital eversion. Digital eversion of the colon consists of three steps: *center line determination,* *cross-section formation*, and *colon eversion*. Let us assume that positive charges are distributed along the curvilinear centerline, as shown in (Figure 2(b)). At each point on the centerline, a set of electrical force lines can be found which are from that point and locally orthogonal to the centerline. The curved cross-sections based on these electrical force lines cover the space around the inner surface sufficiently and consistently, even for highly curved colon segments (Figure 2(b)) [10].

**Figure 3:**
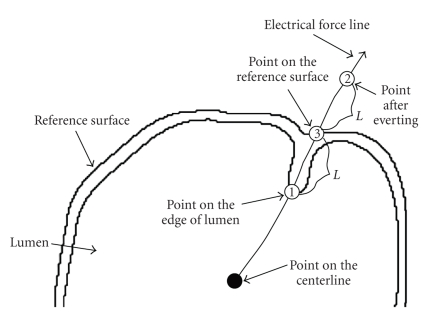
Everting process with respect to an electrical force line, which is from a centerline point in a curved cross-section. Along the force line, the length of a curved segment from point (1) to point (3) equals the length of a curved segment from point (3) to point (2).

To reduce the computational cost, only charges near the current point on the centerline are used to form the cross-section. We call the corresponding field a partial electrical field. If only one charge is used, planar cross-sections are generated. The partial electrical field method is a tradeoff between the performance and the efficiency [10]. An equiangular sampling scheme [10] can be used to form a cross-section. Let *C*_*i*_ and *F*_*ij*_ be curved cross-sections and an electrical force line, where *i* and *j* are the indices for the cross-section and the line, respectively. Denote *M*, *R*, and *E* as the mucosal surface, the reference surface, and the everted surface, respectively. Let *m*_*ij*_ stand for intersection of *F*_*ij*_ and *M*, *r*_*ij*_ stand for intersection of *F*_*ij*_ and *R*, and *e*_*ij*_ stand for intersection of *F*_*ij*_ and *E*. To determine *e*_*ij*_, our criterion is that the length of a curved segment from *m*_*ij*_ to *r*_*ij*_ on *F*_*ij*_ equals the length of a curved segment from *e*_*ij*_ to *r*_*ij*_ on *F*_*ij*_. The eversion process along one electrical force line is illustrated in Figure 3. After all *e*_*ij*_ are found, the everted surface can be formed and rendered.

The everted surface *E* is larger than the mucosal surface *M* in terms of area, since the distance from *E* to the centerline is greater than the counterpart for *M*. Thus, polyps would appear larger after eversion than the original. To minimize the distortion of the polyps, all *e*_*ij*_ should be scaled down along the electrical force lines with an appropriate factor, forming a surface denoted as *S*. Let *s*_*ij*_ stand for the intersection of *F*_*ij*_ and *S*. We can so determine *s*_*ij*_ that ∑_*j*=1_^*K*^ ∑_*i*=1_^*N*^(dist(*s*_*ij*_, mid(*s*_*ij*_, *m*_*ij*_)) + dist(*m*_*ij*_, mid(*s*_*ij*_, *m*_*ij*_))) is minimized, where dist(*a*, *b*) is the distance between *a* and *b* measured along the electrical force line, mid(*c*, *d*) the midpoint between *c* and *d* measured along the electrical force line, *K* and *N* are the number of the cross sections and the number of the electrical force lines on a cross section, respectively. This particular correction is to scale down the everted surface *E* to *S* so that *S* and the mucosal surface *M* are matched as closely as possible. Note that the scaling of *e*_*ij*_ to *s*_*ij*_ is approximately uniform.

### 2.2. Surface-based eversion

**Figure 4:**
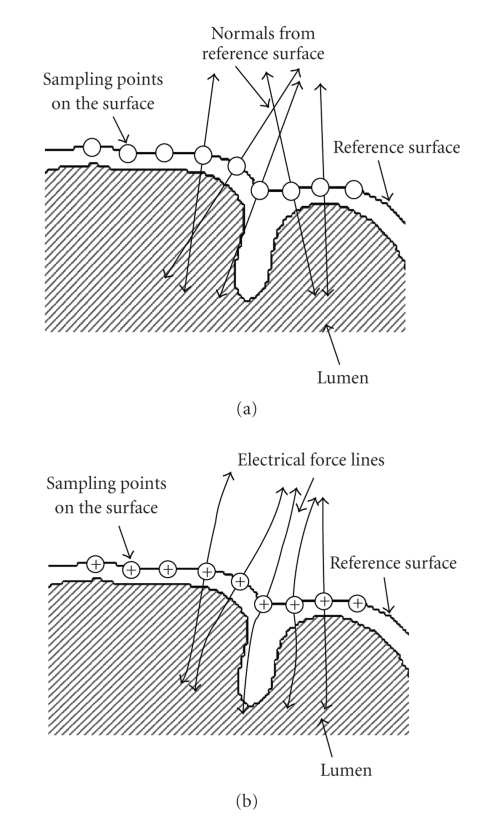
Normals and electrical force lines from sampling points on the reference surface. (a) Normals at different sample points may intersect, which cause distortions in the eversion, and (b) electrical force lines from sampling points on the reference surface, which are locally orthogonal to the surface but are never in conflict.

The surface-based eversion is not only suitable for tubular structures but also for nontubular hollow structures. In this setting, the centerline is no longer required. Instead, the reference surface plays a more critical role. One simplest scheme for surface-based eversion is to implement data reflection along each normal on the reference surface. Calculating normals can be done in the three steps: (1) extracting triangular elements based on the reference surface; (2) calculating normals for all the triangular elements; (3) for each point on the reference surface, estimating the normal from the normals of the neighboring triangular elements. Note that normals derived from different points on the reference surface may intersect, as shown in Figure 4(a). Distortions may occur if the everting operation is done along these normals.

Another surface-based eversion is based on electric force lines, similar to what has been discussed in the preceding subsection. This time, positive charges are distributed over the reference surface. At each sampling point on the surface, an electrical force line, which is locally orthogonal to the surface, is generated. According to electrical field theory, these lines never conflict (Figure 4(b)).

Similar to the centerline-based eversion, the partial electrical field around each point on the reference surface can be formed, that is, charges are only assumed in a prespecified neighborhood of that point to generate the electrical force line. The next steps have only minor change in comparison with the centerline-based eversion counterparts.

**Figure 5:**
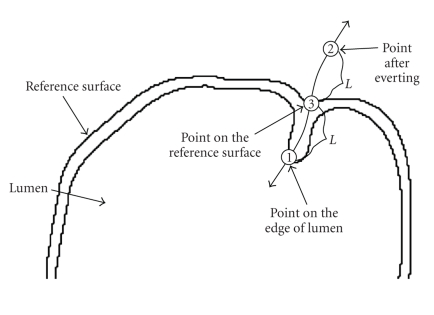
Everting process for a point on the reference surface. Along the curved electrical force line, the length from (1) to (3) equals the length from (3) to (2).

Let *F*_*j*_ be an electrical force line, where *j* is the index. The definitions of *M*, *R*, *E*, and *S* remain the same. Let *m*_*j*_ stand for the intersection of *F*_*j*_ and *M*, *r*_*j*_ for intersection of *F*_*j*_ and *R*, and *e*_*j*_ for intersection of *F*_*j*_ and *E*. The criterion for determining *e*_*j*_ is that the length of a curved segment from *m*_*j*_ to *r*_*j*_ equals the length of a curved segment from *e*_*j*_ to *r*_*j*_ on *F*_*j*_. The eversion process along one electrical force line is illustrated in Figure 5. Again, all *e*_*j*_ should be scaled down along the electrical force lines with an appropriate factor to form the surface *S*. Let *s*_*j*_ stand for intersection of *F*_*j*_ and *S*. We can so determine *s*_*j*_ that ∑_*j*=1_^*P*^(dist(*s*_*j*_, mid(*s*_*j*_, *m*_*j*_)) + dist(*m*_*j*_, mid(*s*_*j*_, *m*_*j*_))) is minimized, where *P* is the number of the electrical force lines.

## 3. RESULTS

We used the patient dataset acquired at the Walter Reed Army Medical Center, USA (http://www.gris.uni-tuebingen.de/areas/scivis/volren/datasets/new.html, Study 289 (213), Colon Prone). It was obtained from an abdominal spiral CT scan with prone orientation after colon cleansing and insufflating. The image volume was made 512 × 512 × 463 voxels of 0.625 × 0.625 × 1.0 mm. The dynamic range was compressed into 256 grey-levels. The mucosal surface of the colon was segmented via a regional growing algorithm. Two simulated hemi-ellipsoidal polyps of semi-axis lengths 4, 4, and 6 mm were digitally implanted.

The segmentation and eversion algorithms were implemented in the Visual Studio platform (Microsoft Corporation) with the C++ language and ITK library (Insight Segmentation and Registration Toolkit (http://www.itk.org/index.htm)). The surface rendering was done using the VTK (Visualization Toolkit (http://www.vtk.org)).

**Figure 6:**
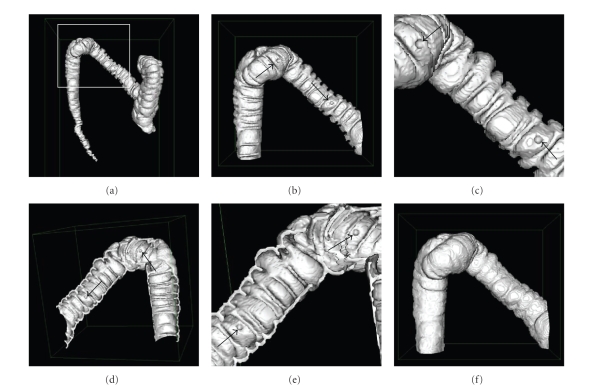
Surface rendering of the original colon lumen with two digitally implanted polyps and its reference surface for digital eversion. (a) An outside view of the whole colon lumen, in which the portion defined by the frame is selected for the digital eversion, (b) a magnified view of the selected colon segment and the simulated polyps (arrows), (c) a magnified view of (b), (d) a bisected colon to show the inner surface and the simulated polyps (arrows), (e) a magnified view of (d), and (f) the reference surface for the digital eversion.

A small portion of the colon lumen was chosen for digital eversion (Figure 6(a)). It contained the transverse and descending colon segments and hence had a high curvature (Figure 6(b)). To present the inner surface of this portion better, a cut plane was used to “open” the colon, as shown in Figure 6(d). Figure 6(f) shows the reference surface derived from dilating the lumen with a spherical kernel.

**Figure 7:**
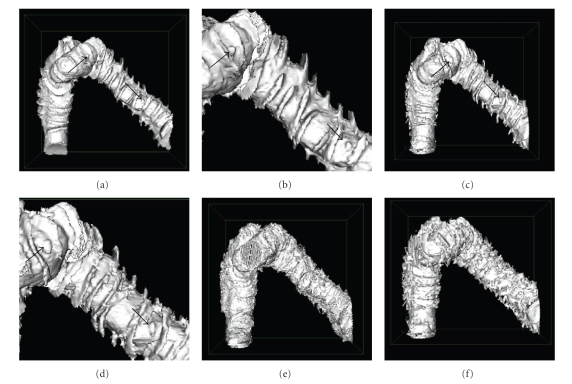
Digital eversion results using representative methods. (a) The centerline-based eversion using the partial-electrical-field method, (b) a magnified view of (a), (c) the surface-based eversion using the partial-electrical-field, (d) a magnified view of (b), (e) the everted mucosal surface based on the planar cross-sections orthogonal to the centerline, and (f) the mucosal surface everted along the normals of the reference surface.

Figure 7(a) shows the digital eversion using the centerline-based partial-electrical-field method (with 181 neighboring charges). As a result, the mucosal surface of the colon can be visualized from outside. Figure 7(c) demonstrates the surface-based eversion using the partial-electrical-field method, in which the sampling points in a 31 × 31 neighborhood were assumed on the reference surface. For comparison, Figure 7(e) is the eversion outcome based on the planar cross-sections locally orthogonal to the centerline, while Figure 7(f) is the eversion result along the normals of the reference surface. Like in the case of unfolding the colon with planar sections [10], both Figures 7(e) and 7(f) exhibit noise and artifacts, making the polyps difficult to be detected.

## 4. DISCUSSIONS AND CONCLUSION

The purpose of this paper is to present a new idea, digital eversion of a hollow structure, and demonstrate its feasibility. Indeed, we have demonstrated that the eversion methods seem to be performing complementary or superior to the existing methods for visualization of hollow-structures. This approach makes it possible to view the inner surface of a hollow structure from outside, while keeping relative positions of key features and the global picture basically unchanged. Although in this paper we used the colon eversion as an example, the technique can be applied to any other hollow structures such as gastrointestinal tract, respiratory tract, urinary tract, blood vessels, spine, and so on, and even solid organs.

The centerline-based method is more efficient but it can only be applied to tubular structures and is sensitive to the accuracy of the centerline determination. The surface-based method can be applied to any hollow structure but it is more time-consuming. Better eversion results are expected when a hollow structure has a smoother inner surface such as blood vessels, bladder, uterus, and so on. The eversion with respect to planar cross-sections or with surface normals is the most straightforward, and may be applied to a tubular organ with a smooth inner surface, no folds, and little flexure; such as esophagus, oviduct and urethra.

Much work is needed for refinement of the algorithms and optimization of the parameters. Possibilities include, but are not limited to, collaboration with radiologists to do extensive reader studies (using more datasets) with actual polyps, utilization of new techniques such as conformal mapping, finite element methods and area-preserving mapping [13–19, 23–26] to obtain better eversion results, and integration with other visualization methods; for example, a tortuous tubular structure may be first straightened completely or to a certain degree, and then be everted. When certain segments block the view of other segments, we may remove the front segments to get a good view for certain areas. The morphology filter with the varied size should be adopted depending on the size of the haustras and folds and the curvature of the colon.

In conclusion, we have conceptualized a new idea on digital eversion of a hollow structure. Practical algorithms have been developed according to the electrical field model, which gives electrical force lines from either a charged centerline or a charged reference surface. The preliminary experiments have demonstrated that the eversion of a hollow structure is feasible and promising for inspection of hollow structures, especially in medical imaging applications.
